# Impact of a Mindfulness‐Based Intervention on Pain and Psychological Factors in Women With Chronic Painful Temporomandibular Disorders

**DOI:** 10.1111/joor.70028

**Published:** 2025-08-06

**Authors:** Melissa de Oliveira Melchior, Laís Valencise Magri, Kranya Victoria Díaz‐Serrano, Elton Brás Camargo Júnior, Felipe Fregni, Kevin Pacheco‐Barrios, Riccardo Lacchini, Christie Ramos Andrade Leite‐Panissi, Edilaine Cristina Silva Gherardi‐Donato

**Affiliations:** ^1^ Ribeirão Preto College of Nursing, Center of Mindfulness and Integrative Therapies ‐ CEMITI University of São Paulo Ribeirão Preto São Paulo Brazil; ^2^ Ribeirão Preto School of Dentistry University of São Paulo São Paulo Brazil; ^3^ Rio Verde University Rio Verde Brazil; ^4^ Spaulding Modulation Center Spaulding Rehabilitation Hospital Boston Massachusetts USA; ^5^ Harvard Medical School Boston Massachusetts USA; ^6^ Department of Epidemiology Harvard T.H. Chan School of Public Health Boston Massachusetts USA; ^7^ Vicerrectorado de Investigación, Unidad de Investigación Para la Generación y Síntesis de Evidencias en Salud Universidad San Ignacio de Loyola Lima Peru; ^8^ Philosophy, Science and Letters of Ribeirão Preto University of São Paulo São Paulo Brazil

**Keywords:** biopsychosocial model, chronic pain, facial pain, mindfulness, pain experience, pain‐related TMD

## Abstract

**Background:**

Psychosocial factors influence the perception of TMD signs and symptoms. Regular mindfulness practice supports their management through emotional and cognitive self‐regulation, modulating the TMD‐related pain experience.

**Objective:**

To analyse the influence of a mindfulness‐based intervention on pain, cognitions, behaviour and emotions reported by women with TMD‐related chronic pain.

**Methods:**

Eighty‐four women, aged between 18 and 61 years, with chronic TMD (diagnosed according to the Diagnostic Criteria for Temporomandibular Disorders, DC/TMD), were randomised into an intervention group (IG) or a control group (CG). The intervention group underwent an 8‐week mindfulness practice programme, consisting of weekly 2‐h sessions and one immersion session lasting 4 h. The entire programme consisted of nine meetings. The control group did not receive any intervention during the same period. All participants underwent clinical pain assessments and completed self‐administered questionnaires before and after the intervention period. These questionnaires addressed cognitive, behavioural and emotional variables. Evaluators were blinded to the group allocations of the participants. Simple linear regression was used for the main statistical analyses, with significance established at *p* < 0.05.

**Results:**

Fifty‐three women completed the study (IG = 30; CG = 23). The intervention had a positive influence on the following variables: number of pain points, pain pressure threshold (PPT), stress, catastrophic thoughts and facets of mindfulness, including ‘observe’, ‘distract’, ‘non‐react’ and overall mindfulness levels (all analyses at *p* < 0.05).

**Conclusion:**

This unique trial, in which the mindfulness‐based intervention was compared against a control (no treatment), showed that mindfulness can contribute to the sensory, cognitive, behavioural and emotional management of pain‐related TMD. The improvement in pain, therefore, was probably influenced by the regulation of psychological aspects related to it.

## Graphical abstract

This study investigates the effects of a mindfulness‐based intervention (MBI) on women aged 18–61 with chronic pain related to temporomandibular disorders (TMD). Eighty‐four participants were randomised into an intervention group (IG) and a control group (CG). The IG received 8 weekly 2‐h MBI sessions plus a 4‐h immersion. Outcomes were assessed through clinical pain evaluations and self‐reported cognitive, behavioural, and emotional questionnaires, both pre‐ (T0) and post‐intervention (T1). Statistical analysis using simple linear regression (*p* < 0.05) revealed significant improvements in pain points, pain pressure threshold (PPT), stress, catastrophic thinking, and mindfulness facets in the intervention group. The findings support MBI as a valuable strategy in managing TMD‐related pain through cognitive and emotional regulation.

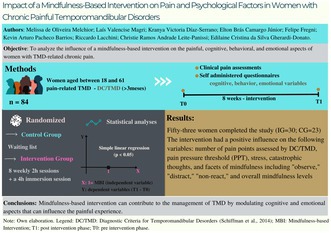

## 1 Background

Temporomandibular Dysfunction (TMD) can be associated with various pain conditions, multisystemic alterations, dysfunctional behaviours and emotions, and it influences social interactions [1, 2]. Subjects with TMD‐related chronic pain are more predisposed to other painful comorbidities such as fibromyalgia, low back pain, neck pain and headaches, among others [1, 2]. TMD‐related chronic pain, which can be associated with widespread pain comorbidities, is associated with physiological alterations in pain processing, which in turn is associated with a higher risk of developing TMD, greater difficulty in controlling the condition and the persistence of signs and symptoms [3, 4]. Previous studies have shown that individuals with TMD‐related chronic pain have a reduced pressure pain threshold (PPT) at bodily points beyond the masticatory muscles than healthy individuals [5]. The perception of TMD signs and symptoms is influenced by psychosocial factors, which are associated with the multidimensionality of pain [6]. The pain modulation process is linked to mechanisms of descending inhibition through the release of neurotransmitters such as serotonin, dopamine and norepinephrine, which control endogenous analgesia [6]. Psychological factors such as pain catastrophising, anxiety and stress can influence this pain control process, increasing nociceptive sensitisation [7, 8]. Women with elevated levels of anxiety and stress are at greater risk of developing myofascial pain, with psychological states of anxiety and stress strongly associated with the diagnosis of TMD‐related pain [9, 10].

Treatment of this complex condition typically involves multiple treatment approaches tailored to each case [2]. In addition to pharmacological [11] or physical peripheral‐level treatments [12, 13], strategies associated with cognitive‐behavioural‐emotional management within a biopsychosocial context are strongly recommended [2]. Mindfulness strategies are considered a valuable tool for this purpose [14]. In the context of chronic pain, the ‘cognitive‐behavioral‐emotional axis’ refers to the interconnectedness of an individual's thoughts (cognitions), actions (behaviours) and feelings (emotions) in relation to their pain experience. These factors are often intertwined and can significantly influence the perception and management of chronic pain conditions [2, 14]. Addressing this axis through interventions like mindfulness can lead to improved pain coping strategies and overall well‐being [14].

Beneficial impacts from mindfulness practices include reducing stress, anxiety and depression and potentially contributing to pain reduction [15, 16]. The potential benefit of using mindfulness for self‐regulation of chronic pain arises from the ability to perceive thoughts, expectations and feelings related to one's body as mental processes, dissociating them from the sensation itself. Enhanced body awareness allows recognition of the impermanence of physical and psychological states, contributing to the reduction of suffering and facilitating self‐regulation of the pain experience [14, 15, 17, 18]. This mechanism is accompanied by clinical, hormonal and neural changes observed in both short‐term and long‐term practitioners, which are conducive to the reduction of central sensitisation, lowering of blood pressure, increase in antibody levels and alleviation of symptoms of anxiety, stress and depression, thereby contributing to chronic pain management [19, 20, 21].

Given these possibilities and considering the low implementation cost as a public policy for managing chronic pain, mindfulness‐based intervention is an interesting approach for treating TMD‐related pain. Supporting this idea, a previous study found an association between higher levels of mindfulness and less intense pain experience in women with TMD [22]. Therefore, we tested the novel hypothesis that mindfulness intervention would result in a decrease in TMD‐related chronic pain and its associated behavioural, cognitive and emotional outcomes. A unique trial was designed in which subjects were randomised into active intervention or no intervention as the control for mindfulness.

## 2 Methodology

This is a randomised controlled trial with two arms. The trial identification is ReBEC UTN code: A 25481097800.

### 2.1 Ethical Aspects

This study was approved by the National Research Ethics Council (CONEP) of the Brazilian Ministry of Health (CAAE: 98129918.6.0000.5407), and the volunteers signed an informed consent form.

### 2.2 Inclusion Criteria

Women aged between 18 and 65 years, with a clinical diagnosis of chronic painful TMD (pain for 3 months or more) according to the Diagnostic Criteria for Temporomandibular Disorder (DC/TMD) (Axis I) [23].

### 2.3 Exclusion Criteria

Individuals who had been receiving treatment for painful TMD for less than 3 months were excluded to minimise potential bias. Participants who had recently started treatment (less than 3 months) may still be experiencing the initial effects of their treatment, making it difficult to isolate the specific effects of the mindfulness‐based intervention. Also excluded were individuals with a previous history of tumours, trauma or head and neck surgeries in the prior 6 months, individuals with acute mental disorders or conditions that compromised communication and pregnant women.

### 2.4 Sample Composition and Collection Times

Considering an estimated incidence of TMD of 4% [1, 2] and a margin of error of ±2% with a confidence level of 95% (2), the formula for estimating the sample size [*n* = Z2 × *p* × (1 − *p*)E2n = E2Z2 × *p* × (1 − *p*)] was adapted using the following variables: Z (critical value for the confidence level—1.96), *p* (estimated incidence of painful TMD in women—0.04) and *E* (maximum tolerable error—0.05). The estimated sample size was 59 participants.

Participants were recruited from a specialised centre for patients with Temporomandibular Dysfunction (TMD) and Orofacial Pain at the University of São Paulo, as well as through a targeted social media campaign directed at women. The online invitation included preliminary questions to assess eligibility based on inclusion criteria, such as age and the presence of classic TMD symptoms. Individuals who expressed interest and met these initial criteria were subsequently invited for an in‐person screening and evaluation in accordance with standardised protocols.

All participants underwent clinical evaluations to diagnose TMD [6] and pressure pain threshold [4] and answered self‐administered questionnaires on sociodemographic data, pain experience [24], stress, anxiety and depression [25], catastrophising of pain‐related thoughts [26], central sensitisation [27] and mindfulness [28]. After meeting the eligibility criteria, completing the clinical assessment and responding to the proposed questionnaires (T0), each volunteer was randomly and restrictively allocated to one of the groups: the intervention group (IG) or the control group (CG). This approach ensured a balanced number of participants in each group. To achieve this, the free software Random Allocation Software 1.0 (https://random‐allocation‐software.software.informer.com/), a Windows‐based programme that facilitates block randomisation by generating allocation sequences, was utilised.

The drawing and the allocation of participants to each group were conducted by a researcher not involved in the assessments or the intervention. The evaluators were unaware of which group the participants were allocated to. The allocation was not double‐blind, as the nature of the proposed intervention did not allow for this approach.

Women in the IG underwent an 8‐week mindfulness programme, consisting of weekly 2‐h sessions and one immersion session lasting 4 h, totalling nine sessions. The CG remained on a waiting list for TMD treatment and did not receive any clinical intervention during the research period. After the 8‐week period, predetermined based on the time required to carry out the mindfulness‐based intervention, the volunteers from both groups underwent all the assessments again, making up the second monitoring moment (T1).

### 2.5 Clinical Pain Parameters

The Diagnostic Criteria for Temporomandibular Disorders (DC/TMD) [23] is a standardised clinical diagnostic instrument used to diagnose temporomandibular disorders. It includes both a clinical examination (Axis I) to diagnose physical signs and symptoms, and a psychosocial assessment (Axis II) to evaluate pain‐related disability and psychological factors. In this study, we utilised the Axis I component for diagnosing TMD in participants, as well as assessing the number of pain regions and pain points during mandibular movements and palpation examination.

*Number of pain points*: Based on the clinical diagnostic instrument DC/TMD [23], the average number of pain regions reported by participants (Question E1) was calculated, as well as the number of pain points with ‘familiar’ characteristics during mandibular movements and palpation examination (Questions E4b and E9), and the number of pain points referenced in the palpation examination (Question E9).

*Pressure pain thresholds (PPT)* were investigated in trigeminal areas (the mean of the values found for the anterior temporal muscles, masseters and TMJ bilaterally) and extra trigeminal areas (the mean of the values found for the upper region of the trapezius muscle, elbow‐lateral epicondyle and medial portion of the knee, bilaterally). The choice of the exact point was made based on the participant's indication of the place of most intense pain, found through exploratory palpation of each region investigated. The PPT was evaluated using an algometer (Kratos‐DDK 20, Ind Bras, LTDA), gradually increasing its compression (0.5 kg/cm^2^/s), with the metal tip of the device positioned perpendicular to the anatomical structure, until this mechanical stimulus became painful for the participant. The volunteer was instructed to indicate the moment of transition from a pressure sensation to a pain sensation, even if minimally painful. At that moment, the participant, as instructed previously, pressed the button on the device connected to the algometer to lock the exact compression that would represent the value of her PPT [4].

### 2.6 Psychometric Instruments

Since pain is considered a multidimensional experience apart from its diagnosis and sensory description, we chose the self‐administered questionnaire ‘Brief Pain Inventory ‐ Brazilian version’ (BPI‐B–Short Form) [24]. The BPI‐B has demonstrated good construct validity, with confirmatory factor analysis confirming two underlying dimensions: pain severity (Cronbach's *α* = 0.91) and pain interference (Cronbach's *α* = 0.87). It also exhibits convergent validity, correlating well with other pain measures such as the EORTC‐QLQ‐C30 pain scale and the McGill Pain Questionnaire [24]. It contains four items referring to the perception of pain intensity and seven items about the perception of pain interference in aspects of daily life. Answers to the questionnaire are requested in scores on a numerical scale that ranges from 0 (no pain/did not interfere) to 10 (the strongest pain you can imagine/totally interfered). The total average (severity and interference) was obtained to characterise the overall pain experience of the sample in the previous 24 h. Higher total scores represent higher levels of negative pain experience.

The ‘Depression, Anxiety, and Stress Scale’ (DASS‐21) is a self‐administered questionnaire that provides parameters related to the psychological states of the participants. This scale measures depression, anxiety and stress. Items were scored on a scale ranging from 0 (did not apply to me at all) to 3 (applied to me very much, or most of the time). The depression scale measures symptoms of dysphoria, hopelessness, devaluation of life, self‐deprecation and lack of interest/involvement. The anxiety scale assesses situational anxiety, skeletal muscle effects, subjective experience of anxious affect and panic attacks. The stress scale is sensitive to levels of chronic nonspecific arousal, difficulty relaxing, nervous arousal and being easily upset/agitated. Each dimension was analysed separately. The Portuguese version of the DASS‐21 has demonstrated acceptable internal consistency, with Cronbach's alpha values of 0.81 for depression, 0.77 for anxiety and 0.79 for stress [25].

The Brazilian Portuguese version of the ‘Pain Catastrophizing Scale’ [(BP)‐PCS] is a self‐administered questionnaire composed of 13 questions. It was used in this study to assess the overall level of pain catastrophising, as well as its specific components: helplessness, rumination and magnification. The patients were asked to respond on the basis of scores varying from 0 (almost never) to 5 (almost always) to describe their thoughts or feelings, with a higher final score indicating a greater level of pain catastrophising. The BP‐PCS has demonstrated good internal consistency (Cronbach's *α* = 0.91 for the total scale, and 0.93, 0.88 and 0.86 for the helplessness, magnification and rumination subscales, respectively) and a stable three‐factor structure in confirmatory factor analysis [26].

Considering that chronic pain affects the nociceptive circuit of the central nervous system, and the presence of central sensitisation can affect the pain experience, the participants' central sensitisation was assessed by using the ‘Central Sensitization Inventory’ (CSI) questionnaires. The CSI is a two‐part questionnaire. The first part (Part A) assesses 25 signs and symptoms related to central sensitisation, with specific statements scored on a Likert scale [range: 0 (never) to 4 (always)]. The total score ranges from 0 to 100, with 40 points or more indicating pain central sensitisation. The CSI has demonstrated strong psychometric properties in the Brazilian population, with a test–retest reliability of 0.91 and a Cronbach's alpha of 0.9127. The confirmatory factor analysis yielded a four‐factor structure, supporting the original English version.

The ‘Five Facet Mindfulness Questionnaire’ (FFMQ‐BR), validated for the Brazilian version by Barros et al. (2014), measures levels of mindfulness in a multidimensional way, considering seven facets: observing; describing (subdivided into positive and negative formulation); acting with awareness (subdivided into autopilot and distraction); nonreactivity and nonjudging; each one with different minimum and maximum values, according to the number of questions that characterise it. The maximum total score is 195 points and the minimum is 39 points, obtained through the sum of the scores achieved in the facets, indicating the maximum and minimum level of mindfulness, respectively. To analyse the results, each facet was considered separately, as well as the global level of mindfulness, calculated by the total sum of scores. This scale is capable of measuring a wide range of dimensions and populations with or without meditation experience. The FFMQ‐BR has demonstrated good internal consistency (Cronbach's *α* = 0.81 for the total scale) and test–retest reliability (*r* = 0.90). The 7‐factor structure explained 49.56% of the total variance [28].

### 2.7 Intervention

The intervention consisted of a mindfulness‐based practice programme called ‘8‐Week Mindfulness Program’ (Appendix A; Table A1). The intervention protocol was standardised by certified instructors from the Center for Mindfulness and Integrative Therapies (CEMITI) at Ribeirão Preto Nursing School, University of São Paulo (EERP/USP), Brazil, and comprised practices aligned with the principles of the Mindfulness‐Based Stress Reduction Program (MBSR) [29] and the recommendations of the *UK Network for Mindfulness‐Based Teachers Good Practice Guidelines for Teaching Mindfulness‐based Courses* (2015) [30].

The Mindfulness Program was developed and organised into eight weekly in‐person sessions, each lasting 2 h and one immersion session lasting 4 h, offered to small groups with a maximum of 10 people. The environment was prepared to facilitate mindfulness learning for the groups, meaning it was calm, quiet, spacious and well‐ventilated. It included chairs and cushions, and the floor was covered with an approximately 3‐cm thick rubber mat, allowing participants to choose where to sit comfortably.

According to the intervention programme, participants were instructed to practise the exercises offered during the in‐person sessions daily on the remaining days of the week. To facilitate learning and adherence to daily practices, printed material was provided with the content covered in each session, written in an informal and easy‐to‐understand language, along with guided audio recordings to assist in meditation practices. The audio recordings were made available to participants via email, WhatsApp and Compact Disc (CD), according to their preference. At the end of each session, participants were also provided with a diary to record their practices. The diary was not used for analyses and served primarily to encourage and organise the incorporation of practices into the participants' daily lives. The 8‐Week mindfulness programme is detailed in Appendix A [31].

### 2.8 Statistical Analyses

The Stata‐Statacorp LLC version 15.0 software was used. Data normality was tested using the Shapiro–Wilk test. According to the results of the normality test, prior to the proposed intervention (T0), to verify possible preexisting differences between the groups, t‐student tests for independent samples were performed for the parametric dependent variables and the Mann–Whitney U test for the nonparametric dependent variables. Sociodemographic data were analysed descriptively, and we reported means and standard deviations for continuous variables (such as age) and frequencies for categorical variables (such as income, education level, marital life, physical activity, pain of time, social life and diagnostics). In addition, baseline (T0) comparisons between the intervention and control groups were conducted for sociodemographic variables, using the Mann–Whitney U test for continuous variables and the chi‐square test for categorical variables.

Simple linear regression analyses were used for continuous outcomes, taking the intervention as a predictive variable and the primary outcomes (number of pain spots, BPI‐B and CSI‐A) and secondary outcomes (DASS, PCS and FFMQ) as dependent variables, represented by the difference found between T1 and T0 assessment moments, which allows considering the individual variability in the responses over time for both groups. A multiple linear regression analysis was conducted for the primary outcome ‘PPT Body’ variable, adjusting for age, social life, time of pain and the preintervention mean of PPT Body, since this was the only variable that showed a statistically significant difference between groups at baseline (T0). The intervention variable was coded as a dichotomous predictor (0 = control group, 1 = intervention group), as appropriate for group‐based comparisons using linear regression. Assumptions of regression models, such as linearity, independence, homoscedasticity and normality of residuals, were thoroughly examined to ensure the validity of the results. The value established to consider a significant interaction between the dependent and independent variables was set at *α* = 0.05. The regression coefficients and their corresponding confidence intervals were reported.

## 3 Results

**FIGURE 1:**
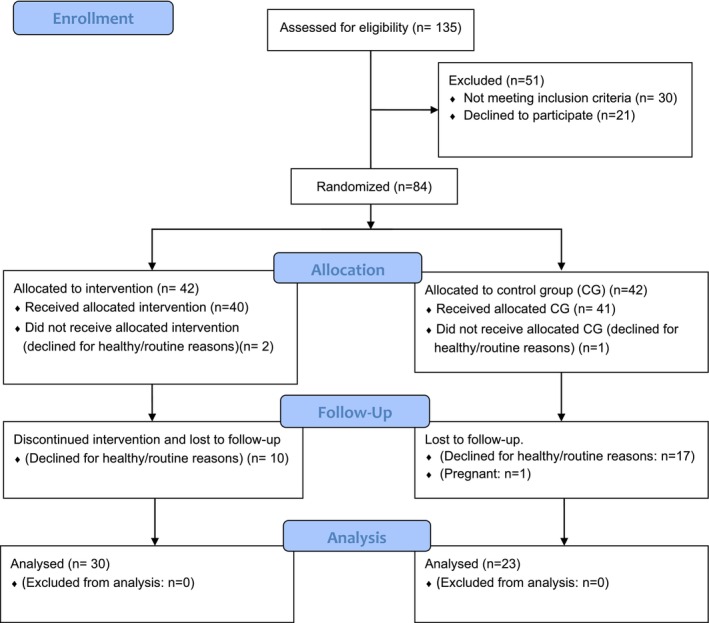
Flowchart: Sample flow throughout the study. *Source:* Adapted from Consort Flow Diagram, 2010.

The estimated sample size would be 59 participants. Considering the practical feasibility of obtaining this number of participants, especially in clinical studies, where uncontrollable factors inherent to the participation of volunteers are present, over a year and a half of project development, the assessment for eligibility was carried out on 135 volunteers, who responded favourably to the public invitation and who agreed to pass by screening the inclusion criteria for the study. Among them, 21 decided to drop out before randomisation. Of the total of 114 women screened, 30 were excluded because they did not meet the study inclusion criteria and 84 were randomly randomised by an online electronic draw (Random Allocation Software 1.0), one by one, between the two research arms, initially composing the intervention group (IG, *n* = 42) and the control group (CG, *n* = 42). The final sample analysed consisted of 53 women with TMD‐related chronic pain [6], 30 in the IG and 23 in the CG, aged between 18 and 61 years. Figure 1 illustrates the flow of the sample throughout the study.

The baseline sociodemographic characteristics and TMD diagnostic of the participants in the two groups are described in Table 1.

**TABLE 1:** Sociodemographic and diagnostic data: Descriptive analyses for both intervention (*n* = 30) and control group (*n* = 23).

	Intervention group	Control group	U Mann–Whitney or Qui‐square test
Age (mean; SD)	34;±6.8	35; ±7.3	0.711
Family income (*n*;%)			0.464
Didn't declare	1; 3.3%	3; 13%	
Until 5 multiples of the minimum wage	19; 63.3%	13; 56.5%	
> 5 multiples of the minimum wage	10; 33.3%	7; 30.4%	
Education level (*n*;%)			0.854
High school completion	6; 20%	3; 13%	
Graduate	18; 60%	15; 65.2%	
Postgraduate	6; 20%	5; 21.7%	
Marital life (*n*;%)			0.268
With partner	15; 50%	8; 34.8%	
Without partner	15; 50%	15; 65.2%	
Physical activity (*n*;%)			0.841
None or occasionally	12; 40%	11; 47.8%	
Once or twice/week	10; 33.3%	7; 30.4%	
Three or more times/week	8; 26.6%	5; 21.7%	
Social life (*n*;%)			0.329
None or occasionally	7; 23.3%	9; 39.1%	
Moderate	21.70%	12; 52.2%	
Very active	2; 6.6%	2; 8.7%	
Pain time (months)	64.20; ±51.36	74.91; ±72.98	0.533
DC/TMD			
Local myalgia	6; 20%	8; 34.8%	0.226
Myofascial pain with referral	22; 73%	14; 60.8%	0.335
Arthralgia	22; 73%	16; 69%	0.676
Headache attributed to TMD	20; 66%	16; 69%	0.823
Disc displacement with reduction	13; 43%	6; 26%	0.194
Disc displacement with reduction, with intermittent locking	0; 0%	1; 4%	0.249
Disc displacement without reduction, with limited opening	0; 0%	0; 0%	—
Disc displacement without reduction, without limited opening	1; 3%	1; 4%	1.00
Degenerative joint disease	1; 3%	1; 4%	1.00
Subluxation	0; 0%	1; 4%	0.434

*Note:* Own elaboration. ‘Multiples of the minimum wage’ refers to the number of minimum monthly salaries. There was no significant difference between the groups for any of the variables.

Abbreviations: DC/TMD: Diagnostic Criteria for Temporomandibular Disorder [6]; SD: standard deviation.

Table 2 presents the baseline (T0) values for all study variables in both the intervention (IG) and control (CG) groups. No statistically significant differences were observed between groups (*p* > 0.05), indicating that they were generally comparable at baseline, with the exception of the variable ‘PPT body’, which showed a significantly higher mean in the control group.

**TABLE 2:** Comparison of study variables between the control (CG) and intervention (IG) groups at baseline (T0). Student t‐test for independent samples (PCS, LDP, BPI, CSI‐A, DC/TMD) and Mann–Whitney U‐test for independent samples (DASS, FFMQ).

Dependent variables	CG (*n* = 23)	IG (*n* = 30)	t‐Test value	p
	Mean (SD)			
CSI_A	53.08 (12.51)	48.46 (12.57)	−1.058	0.295
DC/TMD E1	7.75 (3.42)	7.63 (2.71)	−0.124	0.902
DC/TMD E4b FP	1.25 (1.15)	1.96 (1.73)	1.671	0.101
DC/TMD E9 FP	10.41 (3.47)	11.70 (4)	1.240	0.220
DC/TMD E9 RP	4.70 (3.75)	6.43 (5.01)	1.399	0.167
PCS hopelessness	9.458 (6.67)	9.800 (5.87)	0.591	0.557
PCS magnification	6.37 (4.30)	6.23 (3.66)	0.095	0.925
PCS rumination	10 (4.54)	9.23 (4.50)	−0.406	0.686
PPT body	1.85 (0.80)	1.32 (0.52)	−2.722	0.007^a^
PPT face	0.83 (0.29)	0.72 (0.28)	−1.029	0.308
Total BPI‐B	2.95 (1.94)	3.20 (1.93)	0.487	0.628
Total PCS	25.83 (13.91)	25.26 (12.83)	0.159	0.874
	Median (IQR)		U‐test value	*p*
DASS anxiety	3 (9)	4.75 (7.25)	506.5	0.494
DASS depression	4 (6)	4.00 (9)	475	0.281
DASS stress	9.03 (9)	10.50 (6.75)	473	0.269
Total DASS	16 (22)	18.50 (24)	479.5	0.310
FFMQ_ Act_Aw‐Autopilot	18 (11)	18 (7)	558.5	0.975
FFMQ_Act_Aw‐Distraction	10 (6)	10 (6.5)	508.5	0.510
FFMQ_Describing Negatively	11 (4)	10.00 (4.75)	510.5	0.526
FFMQ_Describing Positively	13 (8)	12.50 (8.75)	547.5	0.865
FFMQ_Non Judging	24 (11)	23 (10.75)	489	0.366
FFMQ_Non Reactivity	19 (10)	15 (7.75)	501	0.452
FFMQ_Observing	26 (11)	28.00 (8.75)	507.5	0.502
Total FFMQ	125 (54)	113 (25.50)	504	0.475

Abbreviations: Act_Aw, act with awareness; BPI‐B, Brief Pain Inventory Brazilian version; CSI‐A, Central Sensitisation Inventory part A; DASS, Depression, Anxiety, Stress Scale; DC/TMD, Diagnostic Criteria for Temporomandibular Disorder; E1, first question of the exam form, from which the number of pain points reported by the participant and confirmed by the examiner was extracted; E4b FP, fourth question of the exam form, from which the number of familiar pain points during maximum unassisted mouth opening was extracted; E9 FP and RP, ninth question of the examination form, from which the number of familiar pain points and the number of referred pain points during palpation examination were extracted; FFMQ, Five Facets of Mindfulness Questionnaire; IQR, interquartile range; Mann–Whitney U‐test, Mann–Whitney test value; Med, median; PCS, Pain Catastrophizing Scale; PPT, Pressure Pain Threshold; SD, standard deviation.

^a^ Levene's test is significant (*p* < 0.05), suggesting a violation of the equal variance assumption.

The descriptive values of the study variables at baseline (T0) and post‐intervention (T1) for both the intervention and control groups are detailed in Table 3.

**TABLE 3:** Descriptive data of study variables at T0 (preintervention) and T1 (post‐intervention) for the Intervention (*n* = 30) and control (*n* = 23) groups. Mean (SD); median (IQR).

Variable	IG T0	IG T1	CG T0	CG T1
	Mean (SD)			
BPI‐B	3.20 (1.93)	2.70 (2.16)	2.95 (1.94)	3.38 (2.21)
CSI‐A	48.46 (12.57)	41.06 (14.61)	53.08 (12.51)	47.60 (14.64)
DC/TMD E1 (freq.)	7.63 (2.71)	5.400 (3.04)	7.75 (3.42)	6.95 (3.23)
DC/TMD E4b FP (freq.)	1.96 (1.73)	1.533 (1.69)	1.25 (1.15)	1.75 (1.56)
DC/TMD E9 FP (freq)	11.70 (4)	10.60 (4.63)	10.41 (3.47)	12.91 (3.52)
DC/TMD E9 RP (freq)	6.433 (5.01)	4.567 (5)	4.70 (3.75)	7.50 (5.45)
PCS hopelessness	9.80 (5.87)	8.63 (4.83)	9.45 (6.67)	11.39 (6.48)
PCS magnification	6.23 (3.66)	4.73 (3.22)	6.37 (4.30)	6.391 (3.67)
PCS rumination	9.23 (4.50)	7.3 (4.44)	10 (4.54)	9.870 (4.60)
Total PCS	25.26 (12.83)	20.66 (11.63)	25.83 (13.91)	27.65 (13.62)
PPT body (Kgf)	1.32 (0.52)	1.54 (0.69)	1.85 (0.80)	1.34 (0.56)
PPT face (Kgf)	0.72 (0.28)	0.84 (0.41)	0.83 (0.29)	3.85 (1.93)
Median (IQR)				
DASS anxiety	4.5 (6.25)	2.0 (4.7)	4.0 (9.0)	3.0 (5.0)
DASS depression	3.5 (8.5)	2.0 (4.8)	6.0 (7.0)	3.0 (5.0)
DASS stress	10.5 (7.5)	6.5 (5.5)	10.0 (8.5)	8.0 (7.5)
Total DASS	18.0 (21.5)	11.5 (14.5)	18.0 (21.5)	12.0 (17.5)
FFMQ act with awareness‐autopilot	18.0 (7.0)	20.0 (3.0)	16.0 (11.5)	17.5 (13.0)
FFMQ act with awareness‐distraction	10.0 (6.8)	10.0 (4.0)	10.0 (6.0)	10.0 (7.5)
FFMQ describing negatively	10.0 (4.8)	12.0 (5.5)	11.0 (3.5)	10.5 (4.5)
FFMQ describing positively	13.0 (8.8)	14.0 (5.0)	14.0 (8.5)	13.0 (7.0)
FFMQ nonjudging	23.0 (10.8)	27.5 (9.5)	23.0 (12.0)	25.5 (10.5)
FFMQ nonreactivity	15.5 (7.8)	20.0 (7.3)	19.0 (9.0)	19.0 (6.8)
FFMQ observing	26.5 (9.5)	30.5 (6.0)	25.0 (8.0)	24.0 (10.5)
Total FFMQ	112.5 (25.5)	134.5 (28.3)	125.0 (57.0)	120.5 (46.8)

Abbreviations: BPI‐B, Brief Pain Inventory Brazilian version; CG, control group; CSI‐A, Central Sensitisation Inventory part A; DASS, Depression, Anxiety, Stress Scale; DC/TMD, Diagnostic Criteria for Temporomandibular Disorder; E1, number of reported pain points; E4b FP, number of familiar pain points during maximum unassisted mouth opening; E9 FP, number of familiar pain points during palpation; E9 RP, number of referred pain points during palpation; FFMQ, Five Facets of Mindfulness Questionnaire; IG, intervention group; IQR, interquartile range; PCS, Pain Catastrophising Scale; PPT, pressure pain threshold; SD, standard deviation.

The regression models were calculated using the delta (Δ) between post‐ and preintervention measures (T1–T0) as the dependent variables. The results of the linear regression analyses revealed significant associations between the intervention and various measures related to pain and the cognitive‐behavioural‐emotional axis of participants with chronic pain related to TMD, indicating a positive effect of the mindfulness intervention on clinical parameters such as the reduction in the number of pain points, increase in pressure pain threshold, reduction in stress levels, decrease in catastrophic thoughts and increase in mindfulness levels (*p* < 0.05). Detailed results of the analyses are presented in Table 4.

**TABLE 4:** Estimates of the effects of the intervention on the change in dependent variables (T1–T0): linear regression analyses (intervention as predictor).

Dependent variable	Coeff (β)	SE	*t*	*p*	95% CI	R‐square adj	F (1,51)	*p* (Prob > F)
BPI‐B total	−0.93	0.64	−1.46	0.151	−2.21, −0.35	0.02	2.13	0.151
CSI‐A	−2.96	2.69	−1.10	0.277	−8.38, −2.45	0.004	1.21	0.277
DASS anxiety	−0.88	1.27	−0.69	0.492	−3.42, −1.66	−0.01	0.48	0.492
DASS depression	−1.84	1.10	−1.66	0.102	−4.05, −0.38	0.03	2.77	0.102
DASS stress	−2.61	1.17	−2.24	0.030*	−4.96, −0.27	0.07	5.00	0.029*
DASS total	−5.33	3.10	−1.72	0.092	−11,55, −0.89	0.04	2.95	0.092
DC/TMD E1 (freq.)	−1.75	0.98	−1.79	0.080	−3.73, −0.22	0.04	3.19	0.080
DC/TMD E4b FP (freq.)	−0.95	0.39	−2.40	0.020*	−1.75, −0.16	0.08	5.76	0.020*
DC/TMD E9 FP (freq)	−4.10	1.21	−3.38	0.001**	−6.53, −1.67	0.17	11.45	0.001**
DC/TMD E9 RP (freq)	−4.43	1.27	−3.5	0.001**	−6.98, −1.89	0.18	12.22	0.001**
FFMQ Act_Aw‐Autopilot	2.12	1.40	1.51	0.137	−0.69, 4.93	0.02	2.29	0.137
FFMQAct_Aw‐Distraction	1.45	0.63	2.30	0.026*	0.18, 2.72	0.08	5.27	0.026*
FFMQ describing negatively	1.29	1.00	1.29	0.203	−0.72, 3.31	0.01	1.67	0.203
FFMQ describing positively	0.72	1.10	0.65	0.517	−1.49, 2.93	−0.01	0.43	0.517
FFMQ nonjudging	2.69	1.85	1.45	0.152	−1.02, 6.42	0.02	2.12	0.152
FFMQ nonreacting	2.86	1.39	2.06	0.045*	0.07, 5.65	0.06	4.23	0.045*
FFMQ observe	4.67	1.32	3.52	0.001**	2.01, −7.33	0.18	12.42	0.001**
Total FFMQ	15.81	5.48	2.88	0.006*	4.79, 26.82	0.12	8.31	0.006*
PCS hopelessness	−3.73	1.49	−2.50	0.016*	−6.73, −0.73	0.09	6.23	0.016*
PCS magnification	−1.76	0.78	−2.25	0.029*	−3.33, −0.19	0.07	5.05	0.029*
PCS rumination	−2.06	0.96	−2.16	0.036*	−3.98, −0.14	0.07	4.66	0.035*
Total PCS	−7.56	2.63	−2.88	0.006*	−12.83, −2.28	0.12	8.28	0.006*
PPT body (Kgf)^a^	0.41	0.18	2.30	0.026*	0.05, −0.76	0.44	6.97^b^	< 0.001**
PPT face (Kgf)	0.32	0.09	3.30	0.002*	0.12, −0.51	0.16	10.87	0.002*

*Note:* DC/TMD: Diagnostic Criteria for Temporomandibular Disorder‐E1: first question of the exam form, from which the number of pain points reported by the participant and confirmed by the examiner was extracted; E4b FP: fourth question of the exam form, from which the number of familiar pain points during maximum unassisted mouth opening was extracted; E9 FP and RP: Ninth question of the examination form, from which the number of familiar pain points and the number of referred pain points during palpation examination were extracted; PPT body^a^: Pressure Pain Threshold—average of the six body points tested (trapezius muscle, epicondyle and internal lateral knee, bilaterally) adjusted by age, social life, time pain and PPT body preintervention variables {^b^F (7, 45)}; PPT face: pressure pain threshold—average of the 6 face points tested (anterior temporal muscle, masseter muscle and temporomandibular joint) BPI‐B: Brief Pain Inventory Brazilian version; CSI‐A: Central Sensitization Inventory Part A; DASS: Depression, Anxiety; Stress Scale; PCS: Pain Catastrophizing Scale; FFMQ: Five Facets of Mindfulness Questionnaire; Act_Aw: Act with Awareness; Coeff (*β*): estimated coefficient; SE: standard error; t: statistics associated with each estimated coefficient; *p*‐value: probability of observing the estimated coefficient if the true relationship between the variables was zero and *p* < 0.05 suggest that the coefficient is statistically significant; CI: confidence interval—indicates the range within which the true value of the coefficient is likely to fall with 95% confidence; R‐square adj—explains the variation in the dependent variable; *F*: test that compares the variation explained by the model with the unexplained variation (residual); *p*‐value (Prob > F): probability of observing an *F* value equal to or more extreme than the calculated value, assuming that the null hypothesis is true. * Statistically significant.

## 4 Discussion

Considering the biopsychosocial framework for addressing chronic musculoskeletal orofacial pain [2, 14], the aim of this study was to investigate how the experience of pain‐related TMD could be impacted by a mindfulness‐based intervention. This intervention aims to address cognitive, behavioural and emotional aspects. This occurs through its mechanisms of development, such as attentional regulation, broadening and deepening of body awareness, emotional regulation and changes in self‐perspective in the presence of this pain condition [32]. From this study, significant associations were observed between mindfulness‐based intervention and various measures related to pain and the cognitive‐behavioural‐emotional axis of participants with chronic pain related to TMD, suggesting that mindfulness intervention may be a promising approach for managing painful TMD, offering a novel therapeutic avenue.

A statistically significant reduction (*p* < 0.05) in the number of familiar pain points during unassisted maximum mouth opening and palpation, as well as a decrease in referred pain points during palpation, was observed during the clinical examination using the DC/TMD [23]. Neurophysiological studies have shown that regular mindfulness practice can modulate the activity of brain regions involved in pain assessment and processing, such as the anterior cingulate cortex, dorsolateral prefrontal cortex and insular cortex, suggesting neuroplastic remodelling that may contribute to descending nociception modulation [19, 33]. In this sense, women who underwent this intervention appear to have developed a greater ability to regulate their pain responses, as participation in the 8‐week mindfulness programme significantly favoured an increase in body and facial PPT (*p* < 0.05), parameters related to central sensitisation and nociceptive pain in cases of chronic TMD [3, 4, 5]. These clinical indicators are relevant to the outcome of ‘pain’ from the perspective of those who experience it, and these changes in clinical indicators could be explained by the applied intervention.

No statistically significant differences were observed between the intervention and control groups regarding self‐reported pain intensity and pain interference, as measured by the BPI‐B instrument. This result suggests that the mindfulness‐based intervention may not have produced measurable changes in these specific aspects of the pain experience. However, it is also possible that the BPI‐B instrument was not sufficiently sensitive to detect subtle variations in pain intensity and interference in this sample [24]. The mindfulness intervention may have had a greater influence on sensory and cognitive‐emotional dimensions of the pain experience—such as modulation of pain perception and reduction of catastrophic thoughts—than on pain intensity itself. Moreover, the reported baseline averages for pain interference were relatively low, indicating that TMD‐related pain may not have had a strong impact on participants' daily functioning. Additionally, the BPI‐B's 24‐h recall period may have been insufficient to capture the dynamic nature of pain over time, and its limited specificity in assessing orofacial function could have constrained its sensitivity to detect post‐intervention changes. Future research should consider using additional or more specific instruments to better capture the multifaceted nature of the pain experience in individuals with TMD.

Mindfulness practice promotes greater body awareness, resulting in better understanding and management of physical sensations, including pain, as well as improved cognitive and emotional control [19, 33, 34]. No significant difference was found between the intervention and control groups regarding changes in central sensitisation, as assessed through self‐reported symptoms. Although we hypothesised that the mindfulness‐based intervention could reduce central sensitisation levels—given its documented impact on the regulation of affective, cognitive and physiological processes involved in chronic pain—our findings did not support this expectation (*p* = 0.277). Central sensitisation is characterised by hyperexcitability of the central nervous system, leading to heightened pain sensitivity and amplification of sensory signals and is known to play a critical role in the chronicity of temporomandibular disorders [3, 4, 27]. Mindfulness‐based interventions have been associated with changes in brain regions implicated in pain modulation, including the prefrontal cortex and anterior cingulate cortex [19, 33], which could hypothetically influence central sensitisation mechanisms over time. It is possible that 8 weeks of mindfulness training is not sufficient to induce measurable changes in central sensitisation symptoms, particularly in individuals with longstanding chronic pain. Additionally, the development of new cognitive and behavioural patterns targeted by mindfulness practices often occurs gradually and may require longer‐term engagement to affect underlying neuroplastic processes [35, 36]. Furthermore, the conscious awareness and recognition of bodily and psychological changes—central to self‐reported measures such as the Central Sensitisation Inventory—may lag behind physiological changes, especially in participants who are new to contemplative practices.

The mindfulness‐based intervention led to a significant reduction in pain catastrophising across all dimensions—rumination, magnification and helplessness (*p* < 0.05)—which may help disrupt the self‐perpetuating cycle of chronic pain in TMD. Catastrophic thinking amplifies pain perception through neural mechanisms involving the amygdala, ventromedial prefrontal cortex (PFC) and anterior cingulate cortex (ACC), while also impairing the brain's ability to regulate pain [26, 37]. This contributes to maladaptive neuroplasticity, increased pain sensitivity and emotional distress. Mindfulness may counter these effects by fostering nonjudgmental awareness of thoughts, sensations and emotions, engaging neural circuits involved in pain modulation and emotional regulation [19, 26, 33].

As the processing of pain involves psychosocial aspects, neural networks are also susceptible to responding to awareness and education about this dysfunctional physiological process [38]. Regular training of these practices activates the ventromedial PFC, which is related to automatic emotional regulation, allowing for the alteration of the course of the affective experience. This is reflected in a reduction in amygdala response, dampening adverse reactions related to stress and the sympathetic nervous system [15, 33, 34]. This process may be responsible for the increase in mindfulness levels and the reduced stress levels and catastrophic thoughts we observed (*p* < 0.05). Training to observe internal events with acceptance and without judgement and consciously responding to the different demands of life implies greater awareness and assertiveness in responses and possibly helps regulate stress and catastrophic thoughts related to pain [19, 21, 26, 37].

The mindfulness‐based intervention led to improvements in several facets of mindfulness, including observing, acting with awareness, and nonreactivity. These outcomes indicate that the programme was effective in enhancing core mindfulness skills, as intended. These facets are closely aligned with attentional regulation and emotional self‐regulation—mechanisms proposed to mediate the effect of mindfulness on chronic pain and psychological distress [19, 28, 29, 33]. The observed improvements in pain and psychological outcomes in the intervention group may thus be attributed, at least in part, to these underlying mechanisms. While detailed exploration of the neural and psychological bases of mindfulness is beyond the scope of this study, our findings are consistent with existing literature suggesting that enhanced mindfulness capacity can lead to reduced stress, fewer catastrophic thoughts and better coping with chronic pain conditions. Taken together, these findings support the use of mindfulness‐based strategies as a complement to traditional approaches in the management of painful temporomandibular disorders, particularly when psychological comorbidities are present. Future studies may build on this work by using mediation models or neurobiological outcomes to more directly examine the mechanisms through which mindfulness exerts its effects.

The ‘Non‐judging’ facet, which reflects self‐judgement of internal experiences, did not show significant improvement after the intervention. This may be explained by the known difficulty beginners face in observing thoughts and sensations without judgement [28]. Additionally, the unchanged levels of depression in women with chronic painful TMD—commonly associated with self‐critical attitudes—may also play a role [7]. As depressive mood states often require longer interventions to improve, the continuation of mindfulness practice over time may gradually contribute to changes in this facet. Future studies are needed to further investigate these hypotheses.

Overall, the findings emphasise the ongoing need for research to identify strategies that promote the comprehensive development of mindfulness skills in clinical contexts, especially in patients with chronic pain conditions such as TMD, so that the extensive neural network involved in its modulation can be stimulated [19, 33, 36]. Mindfulness intervention can complement the array of strategies for managing chronic painful TMD by offering meditative practices with a cognitive–behavioural–emotional focus. Its strategies promote self‐awareness, reduce anxiety related to pain and acknowledge catastrophic thoughts. Consequently, they facilitate control by consciously choosing to direct thoughts towards healthy content and accepting challenging situations as part of the total experience in the present moment, without falling into automatic reactions to unpleasant experiences like pain [2, 16, 19, 33].

The clinical translation of our findings highlights the value of integrating mindfulness‐based interventions into the management of painful TMD within a comprehensive biopsychosocial framework [2, 14]. Effective implementation by orofacial pain specialists begins with a broadened assessment, incorporating screening for psychological factors such as pain catastrophising [26, 37], hypervigilance and emotional reactivity [7, 8, 9, 10], potentially utilising tools like the FFMQ [28]. Key therapeutic strategies involve cultivating a gentle, accepting communication style that encourages patients to observe their experiences without judgement (‘let's simply observe what your body is telling us’) [15, 17, 29, 32] and teaching practical self‐management skills. These skills 21centre on using attentional anchors, such as focusing on the breath and bodily sensations (both general and specifically within the orofacial region), and integrating brief, mindful moments (1–2 min) into daily routines, for instance, during the insertion or removal of oral appliances [15, 29, 32]. Implementation pathways for orofacial pain specialists include pursuing formal mindfulness instructor training [30] or establishing robust referral networks to qualified professionals or group therapy programmes [14, 31], which can facilitate self‐regulation and self‐awareness [32]. Based on the significant improvements observed in psychological factors (PCS, DASS) and pain parameters in our study, this approach is particularly indicated for patients with TMD‐related chronic pain presenting with significant psychological comorbidities [2, 7, 8, 9, 10, 14, 15, 16, 20, 21, 22, 38].

### 4.1 Study Limitations and Future Perspectives

Limitations of this study include several nonspecific factors that may have influenced the results: the lack of an active control group subjected to a sham intervention containing the same nonspecific factors present in the mindfulness intervention, such as guided social interactions by a facilitator, bodily movements and the use of the same environment for both groups. Additionally, the sample size and variation in the number of sessions attended by each participant may have influenced the results. Furthermore, it is worth noting that, although not controllable, participants' previous experience in responding to the questionnaires at T0 may bias their responses at T1, as there may be an awakening to previously unconscious aspects, leading to responses based on self‐observation following this awakening.

Future studies could delineate a programme specifically tailored to individuals with chronic painful TMD, with practices emphasising awareness of the stomatognathic system in the bodily context, harmful oral habits and assessments with instruments contained in Axis 2 of the DC/TMD [23] for the analyses of intervention effects.

## 5 Conclusion

Mindfulness strategies were proposed as a cognitive–behavioural–emotional and sensory intervention for chronic pain related to TMD. Significant changes were observed in clinical parameters. These included a reduction in the number of pain points, an increase in pain pressure threshold, a reduction in stress levels and a reduction in catastrophic thoughts of rumination, magnification and hopelessness. This demonstrates its potential role in the management of painful TMDs. Tailoring the programme with mindfulness practices that emphasise specific aspects of the physical and psychological manifestations of TMDs may result in an even more significant contribution to the management of this chronic pain condition.

## Author Contributions

Melissa de Oliveira Melchior: Conception and design of the research; obtaining the data; tabulation of collected data; interpretation of data; writing of the manuscript. Laís Valencise Magri: Conception and design of the research; obtaining the data; revising the manuscript critically. Kranya Victoria Díaz‐Serrano: Took part in carrying out the intervention, sample recruitment and drafting the manuscript; revising the manuscript critically; contributed to translating the version into English. Elton Bráz Camargo Jr.: Involved in the discussion of statistical analyses; revising the manuscript critically. Felipe Fregni: Involved in discussion, conducting statistical analyses; involved in the review of the English version of the manuscript; revising the manuscript critically. Kevin Pacheco‐Barrios: Involved in conducting statistical analyses; involved in the review of the English version of the manuscript; revising the manuscript critically. Riccardo Lacchini: Conception and design of the research; revising the manuscript critically. Christie Ramos Andrade Leite‐Panissi: Conception and design of the research; revising the manuscript critically; guided the development of the research. Edilaine Cristina Silva Gherardi‐Donato: Conception and design of the research; obtaining financial support and data; interpretation of data; revising the manuscript critically; guiding the development of research. All authors have read and agreed to the published version of the manuscript.

## Conflicts of Interest

The authors declare no conflicts of interest.

## Data Availability

The datasets generated and analysed during the current study are available from the corresponding author upon reasonable request.

## Appendix A

**TABLE A1:** Summary of 8‐week mindfulness‐based intervention programme by week, main topic and learning approach.

Week	Topic	Learning approach
1st	‘Change is easy’	Provides an overview of the programme and lays the groundwork for learning throughout the 8‐week experience. The person starts to recognise that he/she has the necessary tools to be successful in the practice of mindfulness: a body that breathes and a mind that knows, showing that practising mindfulness is something natural and innate.
2nd	‘Breathing is natural’	The expansion of sensoriality and perception is the guideline of this week. The breath represents the central focus of the experience. Feeling the breath, its naturalness, its rhythm, its depth and intervals. By perceiving the breath of the whole body, spaces are opened for the emphasis on feeling and the reduction of verbalisation, suspending the cognitive elaboration of the experience and making it more sensory.
3th	‘Keeping the body conscious’	In this session, the body becomes the research tool in order to reestablish the connection between bodily sensations and mental/cognitive processes, which are part of an integrated unit. The focus is on feeling the body in its entirety, its power and strength as a mode of awareness, and being in the world.
4th	‘Just be’	With curiosity and openness to the full range of experience, this week's recognition and acceptance of the present moment. Simply feeling in the here and now, without judgement or preconceptions, is the field of exploration. Focus and attention will make it possible to be more aware of self‐criticism and limiting thoughts.
5th	‘Moment by Moment’	This session is the moment in the course in which the fundamentals of mindfulness are being laid, and the focus of attention is expanded to perceive the constant impermanence of mental and emotional states. We become more aware of changes occurring moment by moment, in the body, in the breath, in appearance, in experiences, in thoughts, in others, in the environment, in inanimate things. This enables the gradual recognition of transience, fostering the idea of detachment.
6th	‘Welcoming emotions’	This week focuses on developing skills to enhance inner coping resources and support resilience, welcoming and understanding the different emotions associated with comfort or discomfort, pleasant or unpleasant sensations. It enables the recognition of the changing nature of emotions, their transience, nuances and possible associated physical sensations, which helps to foster interpersonal communication and creativity. The recognition of emotions as part of existence itself, creating from emotional experience the bases for the flow of empathy and self‐compassion.
7th	‘Living Mindfulness’	This week, the focus is on recognising the challenges to practising mindfulness in daily life, in exploring the various ways to integrate mindfulness practices with discipline, flexibility and lightness, taking into account the dynamism of life and how circumstances change. With the refinement of attention, it is possible to perceive the beginning, middle and end of mental formations, increasing the awareness of the possibility of choice and decision‐making. Promoting changes in habits and enabling the awakening of skills is the goal of this session.
8th	‘Cultivating Peace’	In this programme‐closing meeting, the retrospective of experiences and lessons learned throughout the course emphasises the foundations built during the 8 weeks. It is the moment to guide and create strategies to encourage sustaining the practices, considering the challenges and the availability of the options. We remind participants that cultivating mindfulness is an ongoing process, involving empathy, compassion and loving‐kindness to promote peace. As we tell participants, the final lesson represents both the end of the programme and the beginning of the rest of your life.

*Note:* Adapted from Gherardi‐Donato et al., 2023 [31].
